# Monosodium Glutamate-Induced Mouse Model With Unique Diabetic Retinal Neuropathy Features and Artificial Intelligence Techniques for Quantitative Evaluation

**DOI:** 10.3389/fimmu.2022.862702

**Published:** 2022-04-27

**Authors:** Yanfei Liu, Hui Huang, Yu Sun, Yiwen Li, Binyu Luo, Jing Cui, Mengmeng Zhu, Fukun Bi, Keji Chen, Yue Liu

**Affiliations:** ^1^ National Clinical Research Centre for Chinese Medicine Cardiology, Xiyuan Hospital of China Academy of Chinese Medical Sciences, Beijing, China; ^2^ Beijing Duan-Dian Pharmaceutical Research & Development Co., Ltd., Beijing, China; ^3^ North China University of Technology, Beijing, China

**Keywords:** type 2 diabetic retinopathy, artificial intelligence, quantitative evaluation, unique diabetic retinal neuropathy features, mice

## Abstract

**Objective:**

To establish an artificial intelligence-based method to quantitatively evaluate subtle pathological changes in retinal nerve cells and synapses in monosodium glutamate (MSG) mice and provide an effective animal model and technique for quantitative evaluation of retinal neurocytopathies.

**Methods:**

ICR mice were subcutaneously injected with MSG to establish a model of metabolic syndrome. We then established a mouse model of type 1 diabetes, type 2 diabetes, and KKAy mouse model as control. The HE sections of the retina were visualized using an optical microscope. AI technology was used for quantitative evaluation of the retinal lesions in each group of rats. The surface area custom parameters of the retinal nerve fiber layer (RNFL), inner plexiform layer (IPL), inner nuclear layer (INL), and outer plexiform layer (OPL) were defined as SR, SIPL, SINL, and SOPL, respectively. Their heights were defined as HR, HIPL, HINL, and HOPL, and the number of ganglion cells was defined as A. Then, the attention-augmented fully convolutional Unet network was used to segment the retinal HE images, and AI technology to identify retinal neurocytopathies quantitatively.

**Results:**

The attention-augmented fully convolutional Unet network increased PA and IOU parameters for INL, OPL, RNFL, and ganglion cells and was superior in recognizing fine structures. A quantitative AI identification of the height of each layer of the retina showed that the heights of the IPL and INL of the MSG model were significantly less than those of the control groups; the retinas of the other diabetic models did not exhibit this pathological feature. The RNFLs of type 2 diabetes were thinner, and the characteristics of retinopathy were not obvious in the other animal models. The pathological changes seen on HE images were consistent with the results of the quantitative AI evaluation. Immunohistochemistry results showed that NMDAR2A, GluR2, and NRG1 were significantly downregulated in the retina of MSG mice.

**Conclusions:**

The MSG retinopathy model is closely associated with neurotransmitter abnormalities and exhibits important characteristics of retinal neurodegeneration, making it suitable for studying retinal neurocytopathies. The AI recognition technology for retinal images established in the present study can be used for the quantitative and objective evaluation of drug efficacy.

## 1 Introduction

It has been observed that long-term diabetes can lead to diabetic microvasculopathy. Diabetic retinopathy (DR) is one of the typical microangiopathies caused by long-term hyperglycemia and insulin resistance resulting in vascular endothelial dysfunction and damage, which can affect vision or even cause blindness in severe cases (1). High-dose streptozotocin (STZ)-induced type 1 diabetes model, low-dose STZ-induced combined with high-fat diet type 2 diabetes model, and other kinds of spontaneous diabetes models mostly present with high fasting glucose, insulin resistance, and dyslipidemia (2). Currently, there is no particularly effective treatment for the early stages of diabetic retinopathy (the early stages of non-proliferative disease). However, retinal neuropathy is the first typical feature that appears early in DR, which is due to the varying severities of lesions in the nerve cells in the retina owing to the action of excessive neurotransmitters and changes in the synaptic transmission of nerve signals, thereby altering the retinal responsiveness to light, and eventually progressing to abnormal microvascular function (3). In contrast, the principle of the traditional models is to completely or partially disrupt the pancreatic function, which does not directly correlate with retinal neuropathy and does not directly damage the retinal nerve cells; therefore, its use in retinal neuropathy and pharmacodynamic evaluation is not compatible.

Monosodium glutamate (MSG) is a salt formed from glutamate and sodium ions and is also commonly consumed. Since the blood–brain barrier of newborn suckling mice is not completely closed, MSG can be converted to glutamate through a series of chemical reactions in the brain after crossing the blood–brain barrier, thereby disrupting the hypothalamic neuron responsiveness and biological functions of tissues and organs that are regulated by the hypothalamus, resulting in a series of metabolic syndrome presentations (4, 5). The major difference between the MSG animal model and other diabetic animal models is that during the juvenile stage, the action of the inhibitory neurotransmitter glutamate results in neuronal cell damage, including damage to the retinal nerve cells. Therefore, it is highly likely that MSG mice have different features compared with other traditional diabetic animal models of DR and have the potential to evaluate retinal neurocytopathy and drug efficacy.

**Figure 1:**
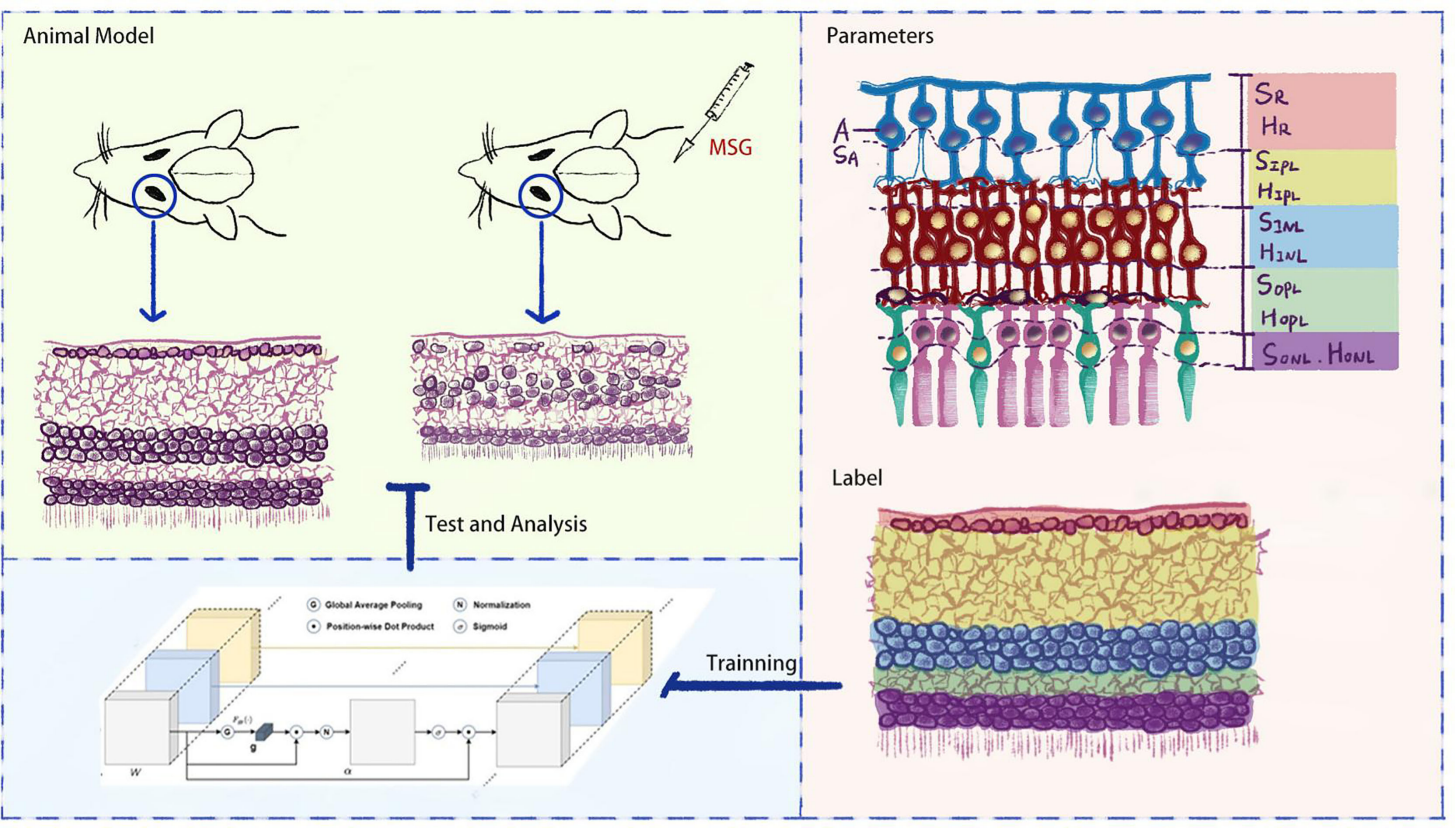
Flowchart of the study.

In order to accurately evaluate the features of DR in MSG mice, this study further expanded the artificial intelligence (AI) recognition zones based on the AI quantitative evaluation method, which was based on retinal hematoxylin–eosin (HE) staining images in previous studies (6) to replace the human eye to observe the subtle pathological changes in retinal nerve cells and synapses in MSG mice, to change the traditional diabetic model for retinal neuropathy, and to provide an important basis for the application of this model on retinal neuropathy and quantitative evaluation of drug efficacy (see **Figure 1**).

## 2 Method

### 2.1 Animal Model and Retinal Sample Preparation

#### 2.1.1 MSG-Induced Metabolic Syndrome Model and Retinal Sample Preparation

All animal procedures were carried out in accordance with the Association for Research in Vision and Ophthalmology (ARVO) Statement for Use of Animals in Ophthalmic and Vision Research and the US National Research Council Guide for the Care and Use of Animals. All procedures involving experimental animals were approved by the University Committee on the Use and Care of Animals (UCUCA) at the Beijing Yizhuang Biomedical Park Animal Center (approval number 2017S007). ICR suckling mice (purchased from HFK Bioscience Co., Ltd., license no. SCXK (Beijing) 2019-0008) were injected subcutaneously with 10% MSG at a dose of 4 g/kg from the second day after birth for 7 days, while suckling mice of the same age, but without MSG injections, were used as the control group. After feeding the suckling mice for 21 days, the pups were weaned and separated into males and females. Further, 15 mice in the model group (MSG mice) and 15 mice in the normal control group were housed in an SPF-grade animal facility with temperature maintained at 22 ± 2°C, relative humidity of 50% ± 5%, 12-h light–dark cycle, and *ad libitum* access to food and water. The body weight, abdominal circumference, fasting glucose, serum triglycerides (TG), and total cholesterol (CHO) were measured at 4, 8, 12, 16, and 18 weeks of age in the mice. The oral glucose tolerance test (OGTT) and insulin tolerance test (ITT) were performed at 8, 12, and 16 weeks. On the day of conducting the OGTT, the mice were fasted; however, they were allowed access to water for 3 h, and 2 g/kg of glucose solution was administered orally after blood was collected at baseline (i.e., fasting blood glucose). The blood was taken from the tail tip at 30, 60, and 120 min after gavage; the glucose level was measured by the glucose oxidase method, and the area under the curve (AUC) was calculated. On the day of the ITT measurement, the mice were fasted without water for 3 h, and 0.4 U-kg^-1^ insulin (Eli Lilly and Company) was injected subcutaneously after the baseline blood was collected and the timer was started. Blood was collected from the tail tip at 40 and 90 min after injection.

The MSG mice were euthanized and dissected when they reached 18 weeks of age; the abdominal fat was harvested and weighed; the adiposity index was calculated (index = weight of abdominal fat/body weight); and eyes were extracted and fixed in 4% paraformaldehyde, paraffin-embedded, cut into sections, and stained with hematoxylin and eosin (H&E). The retinal HE sections were imaged using a light microscope (Olympus BX51, Tokyo, Japan), and the acquisition image format was TIF, with 1,360 × 1,024 and 4,608 × 3,456 pixels, respectively. The same sample retinal sections were used for immunohistochemical staining using 1:500 dilutions of glutamate receptor (NMDAR), neuromodulin (NRG-1), and glutamate (Glu) primary antibodies by using the BCA method. After staining, images were acquired using the same light microscope, and the positivity rates were calculated.

#### 2.1.2 Establishment of Traditional Diabetes Model and Retinal Sample Preparation

Following the previous method (7), a type 1 diabetic rat model was established by intraperitoneal injection of 55 mg/kg STZ into SD rats. A type 2 diabetic rat model was established by intraperitoneal injection of 30 mg/kg STZ twice into SD rats combined with a high-fat diet (10% lard + 20% sucrose + 2.5% cholesterol + 1% cholate + 66.5% conventional chow) during the whole experimental process. The KK-Ay mouse model of spontaneous type 2 DM was established by feeding with a high-fat diet (protein 16.5 kcal%, carbohydrate 37.9 kcal%, fat 45.6 kcal%).

Retinal HE staining was performed on the above models, and the above AI technique was used to quantitatively evaluate the retinal lesions at each layer.

### 2.2 Retinal Image AI Recognition

#### 2.2.1 Custom Parameters and Biological Significance

**Figure 2:**
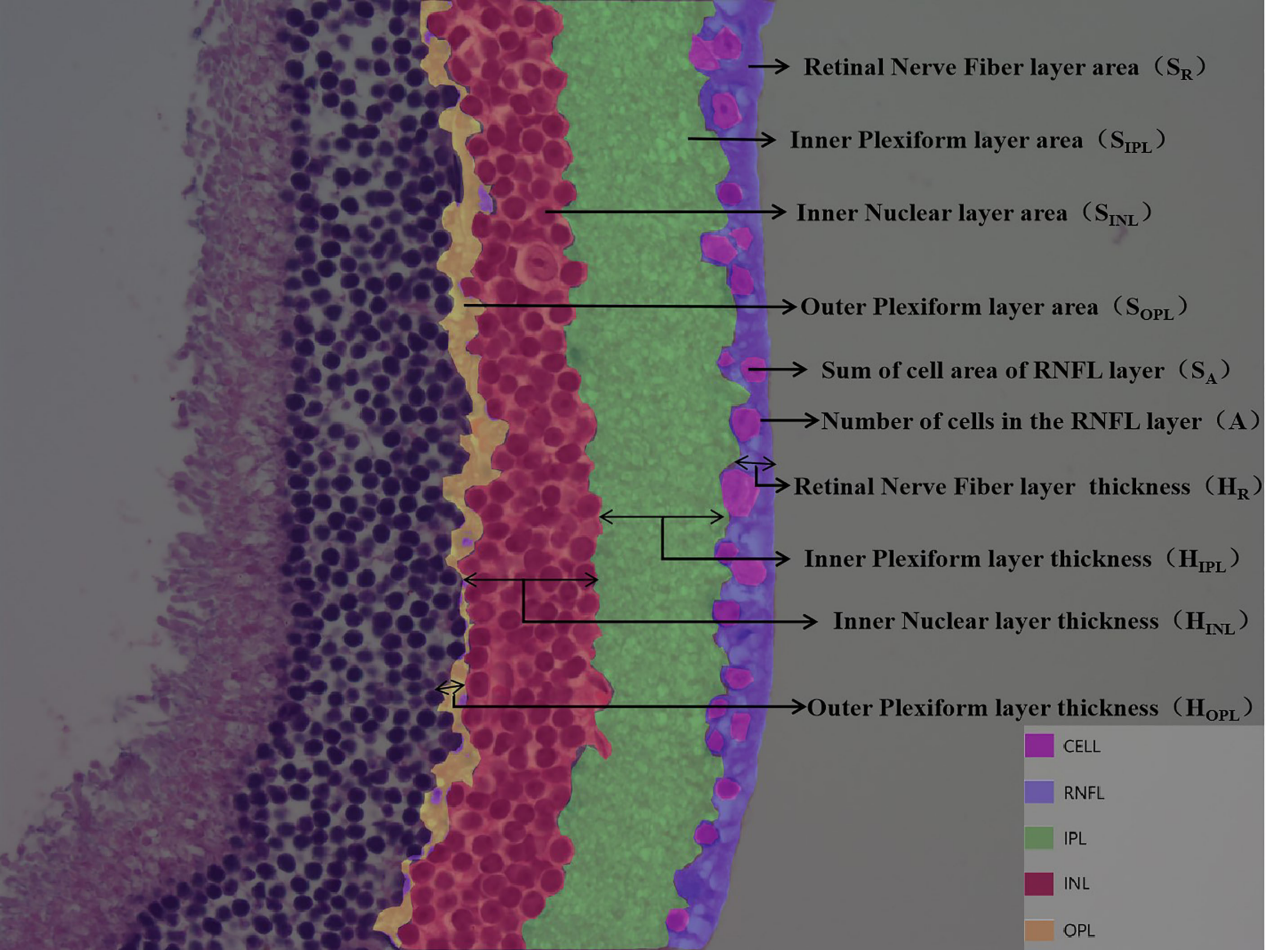
Customize parameters for each layer of retina. DR is divided into non-proliferative and proliferative lesions, of which non-proliferative lesions occur first, and their lesion sites are mainly located in the retinal nerve fiber layer (RNFL), inner plexiform layer (IPL), inner nuclear layer (INL), and outer plexiform layer (OPL). Based on this feature, we artificially defined the RNFL area as S_R_ and the thickness as H_R_; the IPL area as S_IPL_ and the thickness as H_IPL_; the INL area as S_INL_ and the thickness as H_INL_; the OPL area as S_OPL_ and the thickness as H_OPL_; the ganglion cell count as A; and the total ganglion cell area as S_A_.

DR is divided into non-proliferative and proliferative lesions, of which non-proliferative lesions occur first, and their lesion sites are mainly located in the retinal nerve fiber layer (RNFL), inner plexiform layer (IPL), inner nuclear layer (INL), and outer plexiform layer (OPL). Based on this feature, we artificially defined the RNFL area as S_R_ and the thickness as H_R_; the IPL area as S_IPL_ and the thickness as H_IPL_; the INL area as S_INL_ and the thickness as H_INL_; the OPL area as S_OPL_ and the thickness as H_OPL_; the ganglion cell count as A; and the total ganglion cell area as S_A_ (**Figure 2**). The values of S_A_ and A decreased when there was ganglion cell loss or apoptosis; the values of S_IPL_, H_IPL_, S_OPL_, and H_OPL_ decreased when there was vacuolar degeneration or neurosynaptic reduction in the IPL and OPL; and the values of S_INL_ and H_INL_ decreased when there was vacuolar degeneration and neuronal cell loss or apoptosis in the INL.

#### 2.2.2 Full Convolutional Attention Enhancement by Unet Network Construction

**Figure 3:**
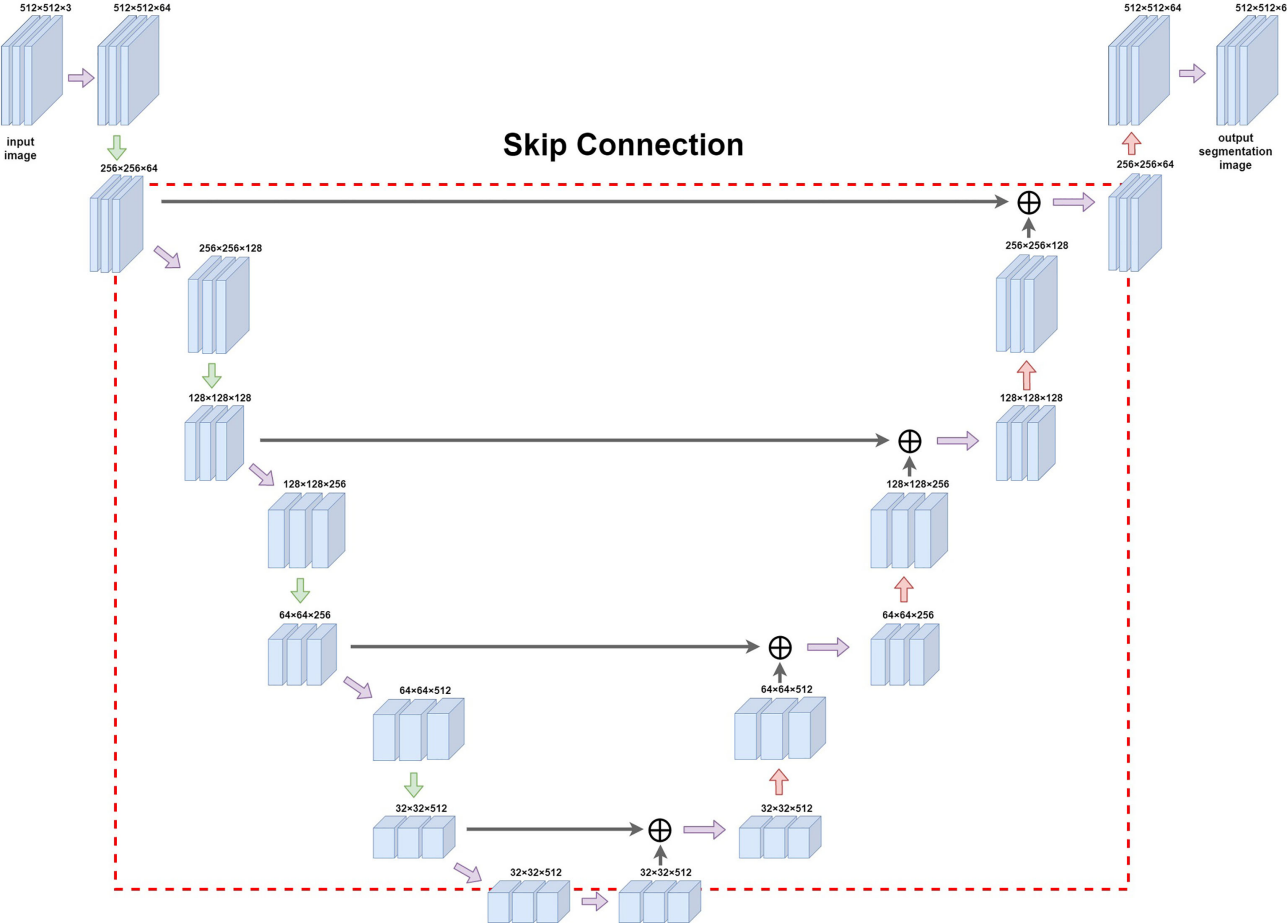
Diagram of Unet network construction. The red box (dashed line) in the figure is the jump structure of u-shaped structure, which can be used to splice and fuse the feature graphs on the left and right sides to deeply fuse the semantic information before and after sampling.

A feature extraction was performed on the input image by using 2D convolution kernels of different sizes: in the downsampling part, we used pooling kernels of size 2 and step length of 2 for twofold downsampling, and upsampling was carried out using transposed convolution for twofold upsampling. The convolution kernel method was used to downsample the image; it uses a sliding window to multiply and add the image pixel values according to position to obtain the eigenvalue at that position, and finally the feature map with all the eigenvalues was obtained. When upsampling with transposed convolution, the matrix with the same shape as the convolution output was transformed to the same matrix as the convolution input to complete the inverse operation of the shape and obtain a feature map with double the size. In order to simultaneously balance the fitting phenomenon owing to the small volume of data and the difficulty of extracting deep semantic information from the shallow network, we chose to build the backbone network (**Figure 3**) by adding the Spatial Group-wise Enhance (SGE) module with VGG-16 as the skeleton. The SGE module first groups the feature maps by channel dimension and performs attention enhancement for each group individually before the group is globally flattened to obtain the vector g. After pooling, g is multiplied with the original group features, normalized, and subsequently activated using the sigmoid function, and finally multiplied with the original group features.

#### 2.2.3 Retinal Image Training and Marker Evaluation

The mouse retinal pathology images were segmented according to the target regions described in Section 2.2.1. Additionally, the segmented results were graphically processed, including pixel thresholding, binarization, erosion operations, and expansion operations, to finally obtain a connected domain for each interval. We labeled the RNFL, IPL, INL, and OPL and ganglion cells in the dataset with a total of 3,500 images, of which 3,000 and 500 images were randomly selected for training and testing, respectively. Prior to the training, we performed a data enhancement to improve the robustness of the model training, including random horizontal flipping with 50% probability, random scaling of images between 0.5 and 1.5x, random RGB to HSV color space transformation, and random rotation by 0°–15°. The VGG-16 model described in Section 2.2.2 was used as the pre-training model for the backbone network, while the SGE module was added for unfreezing training with 100 training rounds, batch size of 4, a learning rate of 0.001, a learning rate decay step of 1, and a decay factor of 0.92. The proposed Unet model was trained and tested using 4 blocks of TiTan X, and Adam optimizer was used to optimize the weights in the network.

The segmentation performance of the model at the pixel level was evaluated using the following 3 metrics.

(1) The pixel accuracy (PA), i.e., pixel-by-pixel calculation accuracy, is given by the following formula:
$$\text{PA}=\frac{\text{TP}+\text{TN}}{\text{TP}+\text{FP}+\text{FN}+\text{TN}}$$
(2) The mean pixel accuracy (MPA), which calculates the average accuracy of all categories, is given by the following formula:
$$\text{MPA}=\frac{1}{\text{k}}(\frac{\text{TP}}{\text{FN}+\text{TP}}+\frac{\text{TN}}{\text{FP}+\text{TN}})$$
(3) The intersection over the union (IOU) ratio, which is the ratio of the intersection of a model’s predicted outcome and the true value for a category to the union of a model’s predicted outcome and the true value, is given by.
$$\text{IOU}=\frac{\text{TP}}{\text{TP}+\text{FP}+\text{FN}}$$
(4) The mean intersection over union (MIOU), the result of the model summation and re-averaging of the ratio of the intersection of the predicted and true values and union of the predicted and true values for each class of outcomes, which is given by:
$$\text{MIOU}=\frac{1}{\text{k}}(\frac{\text{TP}}{\text{TP}+\text{FP}+\text{FN}}+\frac{\text{TN}}{\text{TN}+\text{FN}+\text{FP}})$$

where TP stands for True Positive, FP stands for False Positive, TN stands for True Negative, FN stands for False Negative, and k stands for the number of categories.

### 2.3 Statistical Analysis

The data were statistically analyzed using SPSS 16.0 software, and the experimental data were expressed as x ± SD; additionally, one-way ANOVA was used for comparison between multiple groups, with a difference of *p* < 0.05 considered to be statistically significant.

## 3 Results

### 3.1 MSG Model Characteristics

**Figure 4:**
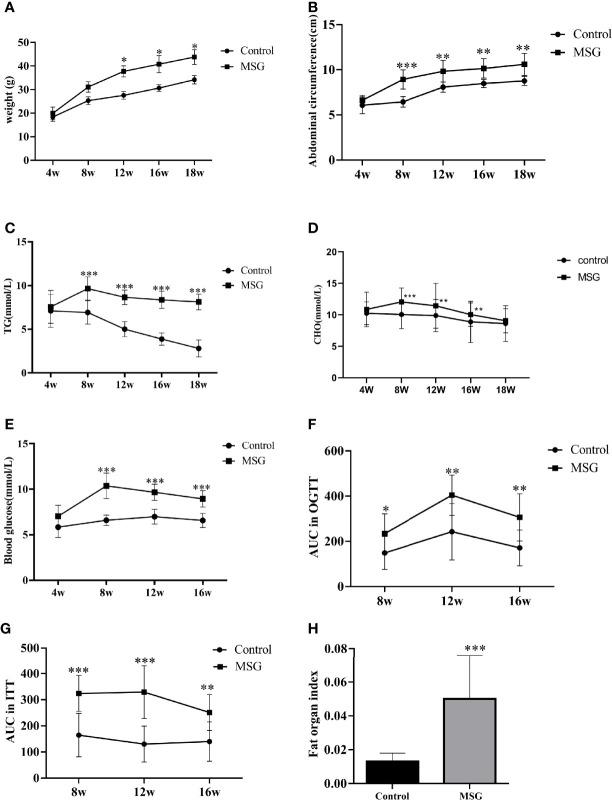
Characteristics of the MSG mouse model. **(A)** Weight change curves of the normal control group and MSG mice. **(B)** Abdominal circumference curve of the normal control group and MSG mice. **(C)** Changes of serum TG in the normal control group and MSG mice at different time points. **(D)** CHO change curves in serum of the normal control group and MSG mice at different time points. **(E)** Changes of fasting blood glucose in the normal control group and MSG mice at different time points. **(F)** Area under OGTT curve (AUC) of oral glucose tolerance of the normal control group and MSG mice at 8, 12, and 16W. **(G)** Area under insulin tolerance (ITT) curve (AUC) of the normal control group and MSG mice at 8, 12, and 16W. **(H)** Fat index of the normal control group and MSG mice at dissection vs. control group: *p < 0.05, **p < 0.01, ***p < 0.001.

MSG-injected suckling mice showed a continuous increase in the body weight and a significant increase in the abdominal circumference compared to normal controls from 4 weeks of age (**Figures 4A, B** **)**. As the age increased, the serum TG and CHO levels were significantly higher in the model group than in the control group (**Figures 4C, D** **)**. The fasting blood glucose was significantly increased in the model group compared with the control group from 8 weeks of age (**Figure 4E**); additionally, the AUC values of OGTT and ITT were higher in the model group than in the control group at different time points (**Figures 4F, G** **)**. At the end of the experiment, the abdominal adiposity index of the model group animals was extremely significantly different compared with the control group (**Figure 4H**).

### 3.2 Retinal Image AI Recognition

**Figure 5:**
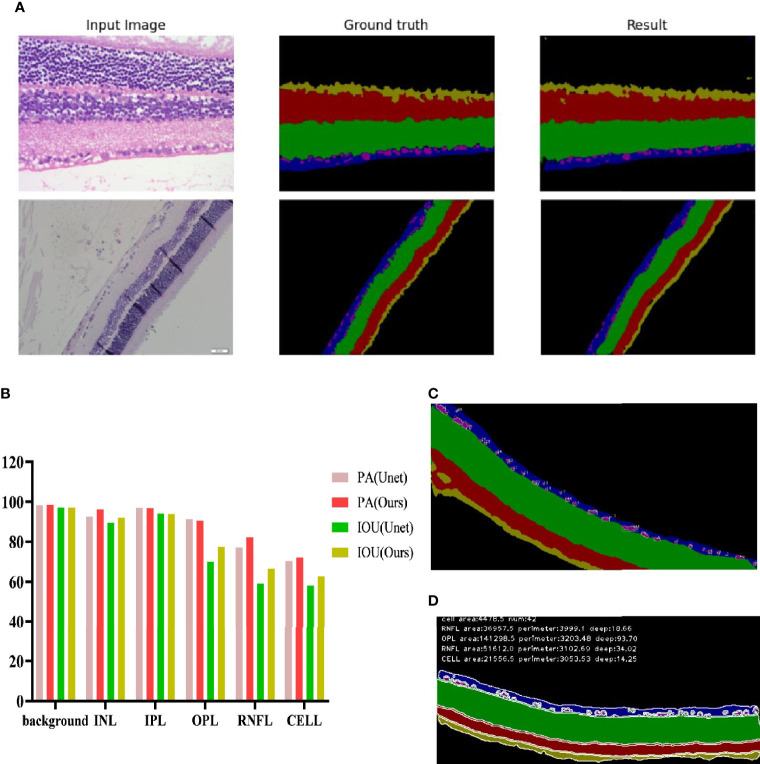
Recognition effect of the Unet network enhanced by full convolutional attention. **(A)** Input image is the collected HE original image; Ground truth is the output result of manually annotated images; Result is the Unet network output Result of full convolutional attention enhancement. **(B)** Background is the Background area outside the target area, and CELL is the ganglion CELL, INL is the kernel layer, IPL is the internal layer, OPL is the external network layer, and RNFL is the nerve fiber layer. PA is pixel accuracy, and IOU is intersection ratio parameter. **(C)** The segmentation result of corrosion calculation was added to the original algorithm. **(D)** Segmentation results obtained by the original algorithm.

The full convolutional attention-enhanced Unet network was used for the segmentation of retinal HE images, and the results showed that the output image segmentation was close to the manual labeling results (**Figure 5A** **)**. Compared with the output results of the conventional Unet network, the full convolutional attention-enhanced Unet network improved the PA and IOU parameters for INL, OPL, RNFL, and ganglion cells, showing that the improved Unet network has a significant improvement over the conventional Unet cross-comparison ratio. Additionally, all three regions have features that are not easily recognized due to the subtlety of the target, indicating that the addition of the SGE module can significantly improve the accuracy in the recognition of some irregular layers and fine structures. For the relatively larger target regions (Background and IPL), the results obtained by the improved Unet network were similar to the traditional Unet network (**Figure 5B**). After obtaining the above segmentation results, we calculated biologically derived parameters on the segmentation map. When calculating the number of cells, as the connected domains generated by segmentation error pixels generated were small, we filtered the overly small cells by erosion operation; for the large adherent cells, we calculated the width-to-height ratio of the outer rectangle by using the connected domain and approximated the number of cells in the adherent region by the width-to-height ratio and summed it (**Figure 5C**). Other target region parameters were calculated by using the above method, and only the connected domains with an area larger than a certain threshold were counted (**Figure 5D**).

**Figure 6:**
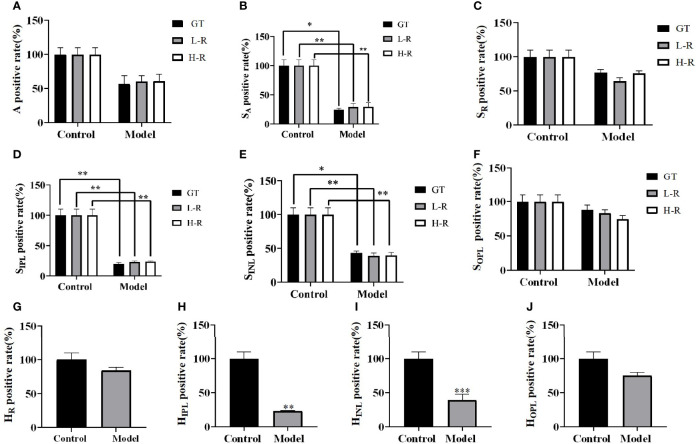
Comparison of the Unet network with artificial interpretation results for full convolutional attentional enhancement. **(A)** Comparative images of positive retinal ganglion cell numbers in control and model group. **(B–F)** Comparative images of the positive rate of the retinal layer area in control and model group. **(G–J)** Comparative images of positive rate of retinal thickness in control and model group. GT is the result of manual interpretation; L-R is the result of low-resolution image AI interpretation; H-R is the result of AI interpretation of high resolution image. The Control group for each method is considered 100% vs. control group: *p < 0.05, **p < 0.01, ***p < 0.001.

To further evaluate the advantages of AI recognition, we measured and calculated the parameters by using the same method for both high-resolution images and low-resolution images and compared the results obtained with the manual method. The manual method used here is pixel measurement by using the histogram function in Photoshop version 2019; this method can only obtain the area of the target region, but not the average thickness of the target region. As seen from the results, for the number of ganglion cells, the results obtained by AI are close to those obtained by manual interpretation (**Figure 6A**), while the results obtained by AI for the two parameters S_A_ and S_INL_ can reflect the difference between the model group and the control group more (**Figures 6B, E** **)**, which also shows that the full convolutional attention-enhanced Unet network is better at detailed structure recognition. For the identification of the S_IPL_ parameters, the results were comparable for the high-resolution and low-resolution images, indicating that resolution has little effect on S_IPL_ (**Figure 6D**). For the S_OPL_ and S_R_ parameters, the results were different for high-resolution and low-resolution images, which also suggests that further research is needed for which resolution to use for the identification of OPL and RNFL target regions (**Figures 6C, F** **)**. The average thickness of the target region can only be obtained by using the AI approach, reflecting the fact that the AI approach can solve problems that are not solvable by the human eye (**Figures 6G** **–J**).

### 3.3 Comparison of Retinopathy Features in MSG Models

**Figure 7:**
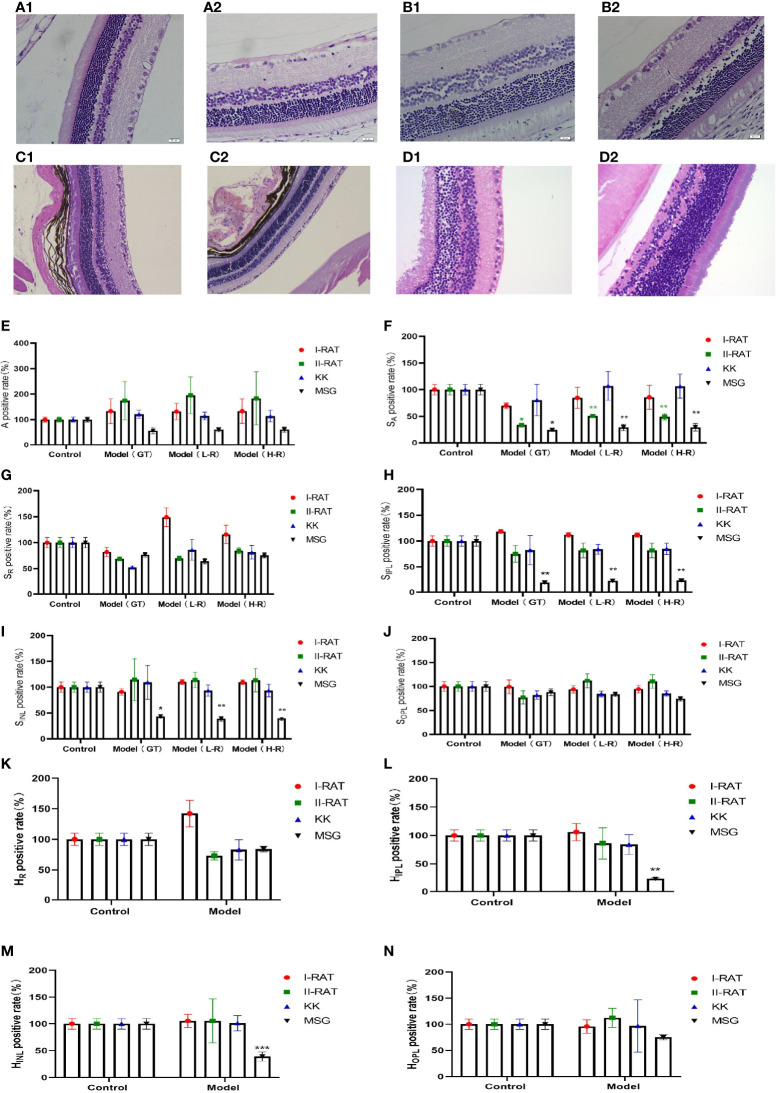
Comparison of the characteristics of retinopathy in diabetic animal models. **(A1)** Retinal HE section images of type 1 diabetic rats. **(A2)** Retinal HE section images of SD rats. **(B1)** Retinal HE section images of type 2 diabetic rats. **(B2)** Retinal HE section images of SD rats. **(C1)** HE section image of C57 mouse retina. **(C2)** HE section image of KK mouse retina. **(D1)** HE section image of ICR mouse retina. **(D2)** HE section image of MSG mouse retina. **(E)** Comparative images of positive retinal ganglion cell numbers in four groups. **(F–J)** The positive rate of the retinal layer area in four groups was compared. **(K–N)** Comparative images of positive rate of retinal thickness in four groups. A is the number of ganglion cells, SA is the total area of ganglion cells, SR is the area of retinal nerve fiber layer (RNFL), SIPL is the area of internal layer (IPL), SINL is the area of kernel layer (INL), SOPL is the area of external network layer (OPL). HR is the thickness of retinal nerve fiber layer (RNFL), HIPL is the thickness of the internal layer (IPL), HINL is the thickness of kernel layer (INL), and HOPL is the thickness of the external network layer (OPL). Red are type 1 diabetic rats, green are type 2 diabetic rats, blue are KK mice, and black are MSG mice. GT is the result of manual interpretation; L-r is the result of low resolution image AI interpretation; H-r is the result of AI interpretation of the high-resolution image. The Control group for each method is considered 100% vs. control group: *p < 0.05, **p < 0.01, ***p < 0.001.

We administered 55 mg/kg STZ intraperitoneally in SD rats to construct a rat model of type 1 diabetes with high fasting glucose; injected 30 mg/kg STZ intraperitoneally in SD rats combined with a high-fat diet to establish a rat model of type 2 diabetes; and incorporated a spontaneous type 2 diabetes KKAy mouse model. Retinal HE staining was performed for each of the above models, and the lesions in each retinal layer were evaluated quantitatively using the AI technique described above. The quantitative results of the RNFL area showed that the type 2 SD rat model was similar to the MSG model; both models had significantly reduced the RNFL area compared with the respective control groups. In the quantitative results of the IPL area, only the MSG model showed a significant reduction, reflecting the typical characteristics that distinguish MSG from other models. The quantitative results of the INL area also reflected the unique thinning features of the MSG model. The quantitative results of the high-resolution and low-resolution images were similar. With regard to the layer thickness, only the IPL and INL thicknesses of the MSG model were significantly thinner compared with those of the control group, while the rest of the retinas of the conventional diabetes model did not show this lesion characteristic (**Figures 7E** **–N**).

From the HE sections, it can be seen that both controls of type 1 diabetic rats and type 2 diabetic rats were normal SD rats with clear retinal layers, intact RNFL, orderly arrangement of ganglion cells, orderly arrangement of INL and ONL cells, orderly IPL and OPL synaptic junctions, and vacuolar degeneration being absent (**Figures 7** **A2, B2**). The HE image results in C57 mice (**Figure 7** **C1**) and ICR mice (**Figure 7** **D1**) showed that the nerve fiber layer was intact, ganglion cells were tightly arranged and orderly, the morphology of the inner reticular layer was intact, the cells of the inner nuclear layer were well-arranged, and intact OPL was visible. In contrast, the RNFL, IPL, and INL of the MSG model were significantly thinner and showed pathological characteristics of neuronal disorder and a reduced number of ganglion cells (**Figure 7D2**). Except for type 2 diabetic SD rats with RNFL thinning features (**Figure 7B1**), the retinopathy characteristics of each model animal were not apparent (**Figures 7A1, C2, D1**), and the pathological changes seen on the HE images of each sample were consistent with the above quantitative evaluation results.

### 3.4 Immunohistochemistry

**Figure 8:**
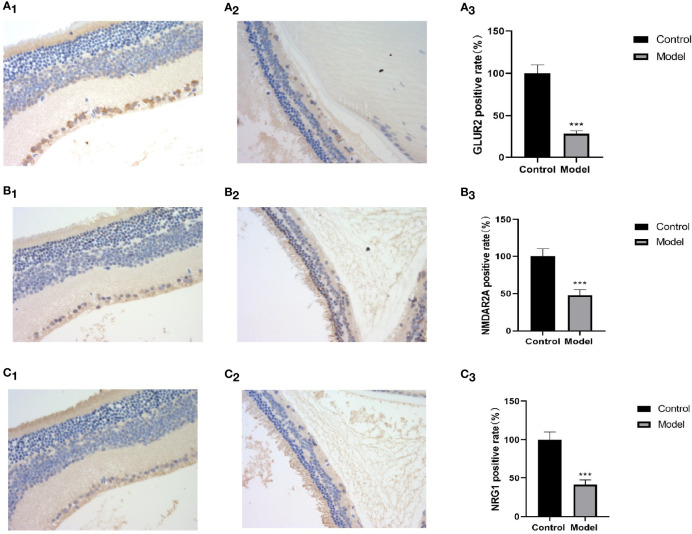
Retinal HE and immunohistochemical images and positive rate calculation. **(A1)** The immunohistochemical image of GLUR2 in normal ICR mice. **(A2)** The immunohistochemical image of GLUR2 in MSG mice. **(A3)** The calculated result of the GLUR2-positive rate. **(B1)** The immunohistochemical image of NMDAR2A of normal ICR mice. **(B2)** The immunohistochemical image of NMDAR2A of MSG mice. **(B3)** The calculated result of the positive rate of NMDAR2A. **(C1)** The immunohistochemical image of NRG1 in normal ICR mice. **(C2)** The immunohistochemical image of NRG1 in MSG mice. **(C3)** The calculated result of the positive rate of NRG1. All HE and immunohistochemical images were taken at ×40. Vs. control group: ***p < 0.001.

Bipolar cells are an important component of the retina, which connect the optic cells to the ganglion cells; additionally, their cytosol is located in the inner nuclear layer, interspersed with ganglion cells in the outer and inner retinal layers and in the nerve fiber layer. The metabotropic glutamate receptor (GluR) and ionotropic glutamate receptor subtype 2A (NMDAR2A) are present on the bipolar cells to ensure the transmission of nerve signals to the optic nerve after light stimulation. NRG1 is a neuromodulatory protein that is mainly expressed in neuronal synapses and in glial cells in the brain and affects neuronal synapses, neuronal migration, and neuronal growth and development by regulating the expression of neuronal receptors (NMDAR, etc.) (8). A deficiency of neuronal NRG-1 expression leads to reduced neurotransmission. Normal mouse retinas contain large amounts of NMDAR2A, GluR2, and NRG1 proteins in all the layers (**Figures 8** **A1, B1, C1** **)**, whereas all three of these proteins are significantly downregulated in the retinas of MSG mice (**Figures 8** **A2, A3, B2, B3, C2, C3**). It is speculated that this change is related to the glutamate injection in MSG mice.

## 4 Discussion

A significant part of the biomedical field consists of microscopic images, for which image segmentation and applications at the microscopic scale are research hotspots in the recent years and prove to be a challenging research area. Since medical and biological images usually have a low data volume, non-uniform input scales, and large differences in target segmentation regions, it is difficult to achieve high-accuracy results by using traditional image segmentation methods (9, 10). With the rapid development of deep learning techniques, convolutional neural networks have significantly improved the ability to extract features and can more easily extract and analyze the image features, thus significantly improving the image segmentation results (11). In this paper, the microscopic scale of the retinal HE images of rats and mice is the micron scale, the retina has ten layers of dense structure, and the layers are interlaced with each other with unclear boundaries. This is especially so in early retinopathy where pathological changes are more subtle, which makes it extremely difficult to use AI technology for the recognition of DR pathological HE images.

In order to overcome the above technical difficulties, we have made a series of improvements to the traditional Unet network. As the convolutional neural network has features, such as translation invariance, rotational invariance, and scale invariance (12), it has no effect on feature extraction in the input image. Therefore, it can achieve better segmentation accuracy when sampling the target image, and similar feature segmentation results can be obtained for images of any size, which increases the universality of the method. In consideration of different image pixels acquired in the practical application, we also use the maximum pooling method to compress the images, thus reducing the computational load of the network. In the upsampling process of the target image, we use the transpose matrix of the convolution matrix for convolution, which can transform a matrix with the same shape as the convolution output to a matrix with the same shape as the convolution input, i.e., inverse operation of the shape, and finally obtained a feature map with double the size, which is convenient for subsequent recognition and analysis. Due to the complex structure of the retina, it is difficult to label the images, and it is difficult to obtain retinal samples with different diabetic features, resulting in a limited number of sample libraries. In addition, large backbone networks are often prone to overfitting due to the small amount of data, while it is often difficult to extract deep semantic information from shallow networks. In order to solve the above contradictions, we choose to build the backbone network with VGG-16 as the backbone and add the SGE attention enhancement module, which can carry out multiple convolutions, normalized activation, attention enhancement, and maximum pooling compression processes to finally obtain the original image at 5 scales. The SGE is a lightweight-attention module, whose role is to obtain extremely strong gain in segmentation performance with almost no increase in the number of parameters and computation, thus obtaining features in each layer and reducing noise. Although the semantics of the retinal pathology images of rats and mice are relatively simple and have a fixed structure, and does not require repetition to filter useless information, the features of all retinal layers are important; thus, both low-level features and high-level semantic features need to be given attention by the network. For this reason, we use a U-shaped structure of skip connection that can combine semantic information before and after sampling well enough to achieve better segmentation accuracy.

Therefore, in this paper, we improved on the traditional Unet network and used a full convolutional attention enhancement-based Unet network for the segmentation of retinal pathology images. In this way, a full convolutional segmentation network with convolutional invariance was implemented, a backbone network suitable for the target region was selected for construction, and a multilevel feature fusion segmentation model based on skip connections was constructed. The experimental results of segmentation also show that the full convolutional attention-enhanced Unet network is better than the traditional Unet network, especially as the segmentation accuracy of the OPL, RNFL, and ganglion cells is significantly improved. However, the implementation and application of the above methods have not been reported in similar papers.

The development of DR is a slow and gradual process from the non-proliferative phase to the proliferative phase, and neuronal changes are an early phenomenon of DR (13), while the RNFL, INL and ONL are the “aggregation sites” for various nerve cells. The ganglion cells in the RNFL are the trigger point for photoreceptor signals, while the bipolar cells in the INL are associated with the ganglion cells and transmit signals to the cone cells and rod cells through depolarization and hyperpolarization of the bipolar cells, and finally to the brain *via* the optic nerve. Thus, DR first begins in the RNFL and spreads to the IPL, INL, and OPL layers as the lesion progresses.

High-dose STZ-induced type 1 diabetic rats, low-dose STZ-induced combined with high-fat diet-induced type 2 diabetic rats, and spontaneous type 2 diabetic KKAy mice are some of the most common models used to study diabetes and its complications. These models are modeled by destroying pancreatic tissue or increasing the fat intake, resulting in a complete or relative deficiency in insulin secretion, leading to abnormal blood glucose and/or insulin resistance, which causes intracellular mitochondrial damage (14), abnormal vascular endothelial cell function, excessive inflammation and oxidative stress, and other pathological processes (8, 15), ultimately leading to a disruption of the blood-retinal barrier and retinal vascular leakage (16, 17), by which point the lesion has shifted from the neuronal cells to the vascular endothelium and pericytes. From this perspective, the conventional diabetic animal model is not a model of retinal neurodegenerative disease and the use of a conventional diabetic animal model is more suitable for evaluating the efficacy of drugs to improve vasculopathy than for evaluating the efficacy of drugs to delay retinal nerve cell degeneration.

MSG mice are a type of model in which MSG is administered to suckling mice just after birth, which in turn develop metabolic syndrome in adulthood (18). The principle of this model is that MSG is continuously administered to mice before the blood–brain barrier is closed, and it is metabolized *in vivo* to glutamate, which is one of the important neurotransmitters in the body. Due to the presence of metabotropic glutamate receptors (mGluR) and ionotropic glutamate receptors (NMDAR) on bipolar cells in the retinal INL, which produce depolarizing and hyperpolarizing responses, respectively, in response to light stimulation, neural signaling can be caused by light modulation (19). Due to the early stimulation of MSG mice with excessive glutamate, bipolar cell lesions were evident in the INL layer of their retinas (this is evident in the HE image identification results and IHC results in this paper). At the same time, the dendrites and axons of bipolar cells extend inward and outward, causing pathological changes, such as vacuolation and thinning in the IPL and OPL, which reduced their connections with ganglion cells and rod cells, resulting in the retraction of optic rod cell dendrites (20) and a gradual loss of neuroprotective protein NRG-1 expression, ultimately leading to a series of neurodegenerative lesions in the retina (21). The present study also demonstrated that the retina of each traditional diabetic animal model, except the MSG model, did not show pathological changes associated with neuropathy. Therefore, the MSG model, which has a pathogenesis closely related to neurotransmitter abnormalities and has characteristic retinal neurodegenerative lesions compared with other traditional diabetic models, is more suitable for early DR drugs, as well as for the evaluation of drug efficacy associated with neurodegenerative lesions. Moreover, the fully convolutional attention-enhanced Unet network proposed in this paper solves the bottleneck of the absence of quantitative evaluation methods for RNFL, IPL, INL, OPL, and ganglion cells and replaces the traditional method of indirectly reflecting nerve cell abnormalities in the retina by labeling various characteristic molecules in nerve cells (22, 23). This can be used to achieve a quantitative and objective evaluation of drug efficacy. Since retinal nerve cell degeneration is closely related to a variety of diseases, by combining with the quantitative identification of retinal nerve cell lesions by AI technology, we believe that this model has a broader application prospect in the field of cognitive impairment, Alzheimer’s disease, vascular dementia, glaucoma, and other neurodegenerative pathologies. Retinopathy is a gradual and complex process, especially for neurodegenerative diseases, which shows different characteristics in the course of disease occurrence and development. After the neural fiber layer and ganglion cell parameters were proposed in the early stage, the research group also proposed customized parameters such as IPL, INL, OPL, and ONL in view of the progressive characteristics of such diseases, so as to provide a more comprehensive quantitative evaluation of the progress and drug efficacy of neurodegenerative diseases. At the same time, each additional custom parameter will pose a greater challenge to artificial intelligence identification and calculation, which is also one of the innovations and novelty of this paper, which can provide reference for more researchers. This study also has some limitations. It only observed the application of the established artificial intelligence method in the animal model of diabetic retinopathy but did not observe and compare its application in other kinds of retinal degenerations such as retinitis pigmentosa, or the corroboration in retinal degeneration associated with other pathologies (Alzheimer’s disease, Huntington disease, retinopathy of prematurity), which is one of the directions worthy of further study.

## Data Availability Statement

The original contributions presented in the study are included in the article/supplementary material. Further inquiries can be directed to the corresponding author.

## Ethics Statement

The animal study was reviewed and approved by the Beijing Yizhuang Biomedical Park Animal Center (approval number 2017S007).

## Author Contributions

YL contributed to the experiment design, manuscript revision, and decision to submit for publication and is the corresponding author. YfL, HH, and YwL performed the experiments and wrote the manuscript. YS, BL, MZ, JC, and FB analyzed the data. KC help to revise the manuscript. All authors contributed to the article and approved the submitted version.

## Funding

This work was supported by grants from the Fundamental Research Funds for the Central public welfare research institutes of China Academy of Chinese Medical Sciences (ZZ15-YQ-017, ZZ13-YQ-001-A1).

## Conflict of Interest

HH was employed by the company Beijing Duan-Dian Pharmaceutical Research & Development Co., Ltd., Beijing, China.

The remaining authors declare that the research was conducted in the absence of any commercial or financial relationships that could be construed as a potential conflict of interest.

## Publisher’s Note

All claims expressed in this article are solely those of the authors and do not necessarily represent those of their affiliated organizations, or those of the publisher, the editors and the reviewers. Any product that may be evaluated in this article, or claim that may be made by its manufacturer, is not guaranteed or endorsed by the publisher.

## Abbreviations

AI, artificial intelligence; AUC, area under the curve; CHO, cholesterol; DR, diabetic retinopathy; HE, hematoxylin–eosin; INL, inner nuclear layer; IOU, intersection over the union; IPL, inner plexiform layer; ITT, insulin tolerance test; MIOU, intersection over the union; MPA, mean pixel accuracy; MSG, monosodium glutamate; OPL, outer plexiform layer; PA, pixel accuracy; RNFL, retinal nerve fiber layer; STZ, streptozotocin; TG, triglycerides.
